# Circulating Levels of miRNAs in Canines Associated with Impaired Kidney Function

**DOI:** 10.3390/biomedicines14081822

**Published:** 2026-08-13

**Authors:** Selena K. Tavener, Dennis E. Jewell, Kiran S. Panickar

**Affiliations:** 1 Science & Technology Center, Hill’s Pet Nutrition, Inc., Topeka, KS 66617, USA; selena_tavener@hillspet.com; 2 Department of Grain Science & Industry, Kansas State University, Manhattan, KS 66506, USA; djewell@ksu.edu

**Keywords:** immune, renal, glomerulus, PCR, histopathology, inflammation

## Abstract

**Background:** Mature microRNAs (miRNAs) have been implicated in inflammation and fibrosis and are associated with the pathogenesis of renal dysfunction. miRNAs are single-stranded RNAs approximately 22 nucleotides in length that can posttranscriptionally modify mRNA by base-pairing with its complementary sequences leading to the silencing of mRNA. **Methods:** We assessed circulating levels of miRNAs in the blood of dogs that were clinically diagnosed as having chronic kidney disease (CKD) post-mortem, after the dogs had lived their full lives. Gene expression was measured using a Canine miRNA PCR array from blood samples that were collected at the natural end-of-life necropsy from dogs with CKD (2–17 yr) and control dogs (5–13.5 yr). **Results:** End-of-life pathology reports indicated interstitial inflammation, fibrosis, and thickening of the Bowman’s capsule. Histopathological analysis of H&E-stained renal sections taken from end-of-life samples showed a significantly higher number of healthy glomeruli as assessed morphologically in controls compared with CKD. There was a significant decline in the levels of miRNAs *cfa*-let-7a, *cfa*-let-7c, *cfa*-let-7f, and *cfa*-let-7g in dogs with CKD compared with controls. In addition, there was also a ≥1.5-fold reduction in levels of *cfa*-miR-93, *cfa*-miR-122, *cfa*-miR-200a, and *cfa*-miR-204 in CKD compared with controls (all ns). These microRNAs may have potential anti-fibrogenesis effect and are also down-regulated in rodent models and in *in vitro* mechanistic models of renal fibrosis, leading to increased fibrosis. There was also a down-regulation of *cfa*-miR-16 (−2.76 fold, ns), which is consistent with its reported role in attenuating kidney injury independent of fibrosis. **Conclusions:** Down-regulation of these miRNAs may be indicative of a reduction in their role in attenuating renal function, leading to impaired kidney function and possibly fibrogenesis. Importantly, the circulating miRNAs may serve as non-invasive biomarkers for impaired renal function in CKD. Nutritional interventions that upregulate selected miRNAs may serve as important targets for slowing the progression of CKD.

## 1. Introduction

Chronic kidney disease (CKD) in dogs is an irreversible and progressive decline in kidney function generally associated with structural changes in the kidney [1,2]. The causes of renal functional decline are likely multi-factorial and, importantly, the functional changes may not always parallel the structural changes that occur [3]. There are several indicators of kidney dysfunction, including urine specific gravity, serum levels of creatinine, blood urea nitrogen (BUN), and symmetric dimethylarginine (SDMA) [4,5]. The incidence of CKD increases with aging and approximately 10% of dogs are affected, as seen in referral institutions and it has been reported that dogs with CKD have shorter survival times when compared with cats [3,6].

There is evidence to indicate that miRNAs play an important role in several renal dysfunctions [7,8]. miRNAs are small non-coding, single-stranded RNAs that are approximately 18–25 nucleotides long. They bind to the 3’ untranslated region (3’ UTR) of target mRNAs to either inhibit translation or promote degradation [9], which could suppress or attenuate gene expression. Increasingly, the role of miRNAs in renal dysfunctions is being elucidated, including in patients with lupus nephritis [10,11], renal fibrosis in diabetic nephropathy [12], end-stage renal disease patients [13] or in patients with stage 5 CKD requiring dialysis [14]. Evidence from rodent studies assessing microRNAs in renal ischemia–reperfusion injury in mice [15], diabetic nephropathy in mice [16], and podocyte injury in diabetic rats [17] also indicates an important role of identifying the signature profile of miRNAs associated with renal dysfunction. Further, there are reports of miRNAs that could potentially mediate or delay the progression of CKD from acute kidney injury (AKI) [18,19].

The role of miRNAs in kidney function is complex, as different miRNAs have different functions in its pathogenesis [20]. The complexity is further recognized when any one miRNA purportedly has different functional effects. For instance, miR-192, which affects SMAD/TGF as well as PI3/Akt signaling cascades, has been reported to play a protective role in the development of diabetic nephropathy in most clinical trials (see [21] for review). In the same review, Wan et al. report that in most experimental studies, expression of miR-192 is associated with the pathogenesis of nephropathy. These studies indicate that while each miRNA plays an important role in health and/or disease initiation and progression, assessing a signature profile of subsets of miRNAs in an already known health condition may provide a different insight into miRNAs associated with it. As such, miRNA expression profiles are associated with different cancers, including upregulation of miR-21 and miR-155 and downregulation of miR-145 and miR-205 in breast cancer; upregulation of miR- 21 and miR-31 and downregulation of miR-34 and miR-let7 in colon cancer [22].

Reports of miRNAs in canines with kidney dysfunction are scarce [23,24] and profiling circulating miRNAs in CKD in canines will help identify newer biomarkers and potential therapeutic targets. In the current study, we assessed, retrospectively, the circulating levels of miRNA from blood collected at necropsy from canines that were diagnosed with impaired kidney function in order to identify the differences in the miRNA profile from canines with no known kidney dysfunction. 

## 2. Materials & Methods

### 2.1. Experimental Design

The study was approved by the Institutional Animal Care and Use Committee, Hill’s Pet Nutrition, Inc., Topeka, KS, USA (Permit/approval Numbers and dates of approval: CP117 (10 March 2010), CP555 (28 October 2013), CP678 (17 December 2015), and CP836 (4 December 2018). All blood samples collected at the end-of-life were stored at −80 °C as bioarchived samples. Samples were then used for assessment of circulating microRNA levels in canines with CKD (*n* = 10; 2–17 yr) as well as controls (*n* = 10; 5–13.5 yr). For CKD, since this was a retrospective study, the blood samples for dogs with a clinical diagnosis of impaired kidney function were assessed for blood urea nitrogen (BUN), a biomarker of renal dysfunction. In addition, the end-of-life pathology, conducted by a veterinary pathologist, indicated interstitial inflammation, fibrosis, and thickening of the Bowman’s capsule. No signs of significant renal pathology were reported in the control dogs. Immediately prior to necropsy, after the dogs had lived their full lives, blood was collected and analyzed for circulating concentrations of albumin, calcium, phosphorus, urea, and creatinine by an in-house laboratory (Roche Diagnostics, Cobas 6000 series, c501 module, Indianapolis, IN, USA).

### 2.2. Histopathology

Tissue sections (5 μm) that were formalin-fixed and paraffin-embedded were used in the study for each dog. Hematoxylin and eosin-stained (H&E) renal cortex sections were examined for renal pathology as described previously [25]. Briefly, changes in the morphology of each glomerulus in every selected field (30–35 fields/slide/dog) by the investigator, who was blinded to the groups. A healthy glomerulus was defined as having a well-defined basement membrane, mesangial cells, endothelial cells, podocytes, parietal epithelial cells, as well as Bowman’s capsule. In contrast, if a glomerulus showed morphologic changes indicating disease, including but not limited to atrophy, lack of defined or significant thickening of the basement membrane, some expansion of the mesangial matrix or glomerular lesion, then the glomerulus was not considered ‘healthy’. The percentage of ‘healthy’ glomeruli was then calculated from the total number of glomeruli in each field and an average of healthy glomeruli per field was obtained for every dog. Statistical analysis (*t*-test) was subsequently performed on the number of healthy glomeruli on the averages taken from all Con or CKD dogs. While performing the histopathological analysis, the investigator was blinded to the groups and the identity of the dogs. 

### 2.3. Blood Collection and RNA Extraction

Gene expression was measured in blood from end-of-life samples collected in PAXgene RNA blood tubes (Qiagen, Germantown, MD, USA) immediately prior to necropsy, after the dogs had lived their full lives. Samples were stored at −80 °C as bioarchived samples and total RNA was isolated using the PreAnalytix PAXgene Blood miRNA kit (Qiagen). The Agilent RNA 6000 Nano kit was used to measure the integrity of total RNA using Bioanalyzer 2100 (Agilent, Santa Clara, CA, USA). The Qubit 3.0 Fluorometer was then used to measure the concentration of total RNA using the Qubit RNA BR Assay kit (Thermo Fisher, Waltham, MA, USA). cDNA synthesis was then performed using the miScript II RT Kit (Qiagen).

### 2.4. miRNA Gene Expression Analysis

Changes in gene expression were analyzed using the miScript miRNA PCR Array Dog miFinder array (Qiagen) following the manufacturer’s protocol. Briefly, each 96-well array plate consisted of a panel of 84 genes. The last row of the array plate included two wells containing *C. elegans* miR-39 miScript primers, six housekeeping genes for normalization, and two reverse transcription control (miRTC) wells to monitor the efficiency of the reverse transcription reaction, and two positive PCR control (PPC) wells to detect any irregularities that may inhibit the PCR reaction. The total RNA input for each sample used for reverse transcription was 200 ng. The cycling program was set to 95 °C for 15 min for the Hold Stage, followed by 40 cycles of 94 °C for 15 s, 55 °C for 30 s, and 70 °C for 30 s. A default melting curve analysis was also performed to verify the specificity of the PCR run using the ViiA7 Real-Time PCR system (Thermo Fisher). The Ct cutoff was set to 35 to be biologically meaningful. Data for the present study was normalized to SNORD68 as the housekeeping gene, as SNORD68 showed the least amount of variance in Ct values in control and CKD dogs.

### 2.5. Statistical Analysis

For the PCR array analysis, the raw Ct values were exported to an Excel file to create a table of Ct values. This table was then uploaded to the data analysis web portal at https://geneglobe.qiagen.com/us/analyze (accessed on 13 August 2020). The cutoff Ct value was set to 35. Ct values were normalized based on a manual selection of a reference gene (SNORD68). The ΔΔCt method was used for determining fold change with SNORD68 as the housekeeping gene, as described below in Results. The fold change is the normalized gene expression 2^−ΔΔCt^ in the CKD sample divided by the normalized gene expression 2^−ΔΔCt^ in the control samples. The *p*-values are calculated based on a Student’s *t*-test based on the replicate 2^−ΔΔCt^ values for each gene in the control and CKD groups. The *p*-value is based on parametric unpaired two-sample equal-variance and two-tailed distribution. 

## 3. Results

**Figure 1:**
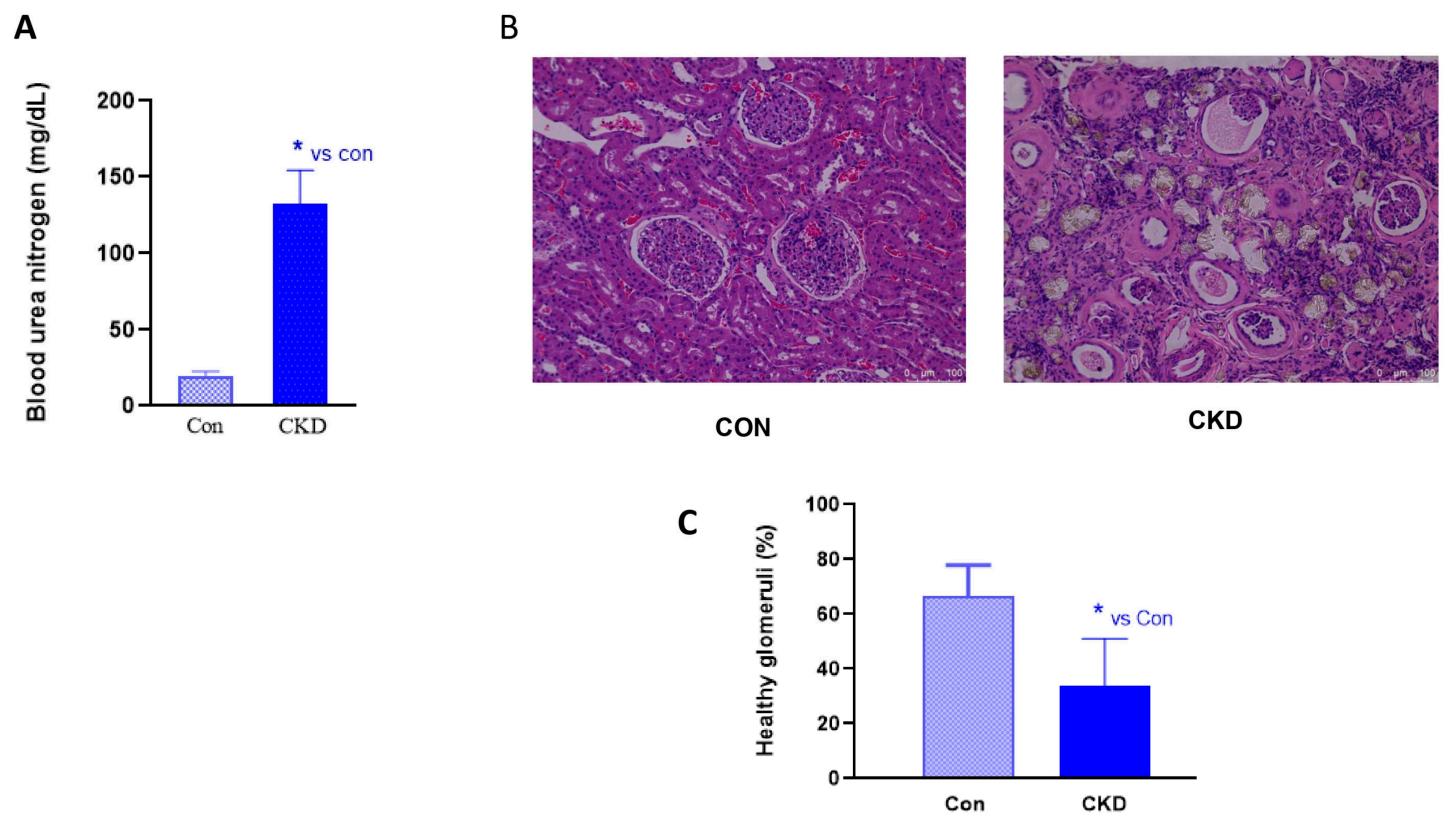
End-points used to characterize control and dogs with impaired kidney function. (**A**) Increased levels of blood urea nitrogen (BUN), as assessed in end-of-life (EOL) samples. Data are presented as mean ± SEM (*n* = 6 Con; *n* = 10 CKD; * *p* < 0.05. (**B**) Representative photomicrographs of H&E-stained renal sections in Control (Con) and chronic kidney disease (CKD). Scale bar = 100 micron. (**C**) Quantification of healthy glomeruli per field in Con (*n* = 10) and CKD dogs (*n* = 10). The number of healthy glomeruli was assessed in 30–35 fields in each slide for each dog (see text for details). Data are presented as mean ± SEM (* *p* < 0.05).

Classification of dogs based on biochemical parameters: Prior to the analysis of the miRNA assessment, dogs were grouped into control and CKD in this retrospective study based on the clinical diagnosis of the veterinarian during life. In addition, at the natural end-of-life, blood samples were collected and a biomarker of renal dysfunction, including circulating levels of creatinine and blood urea nitrogen (BUN), was assessed. Dogs were grouped into control and CKD groups based on the level of circulating BUN, one of the accepted markers of impaired kidney function. There was a significant increase in BUN in CKD dogs (132.4 ± 21.8 mg/dL; *n* = 10) compared with controls (19.33 ± 3.3 mg/dL; *n* = 6; *p* < 0.05; Figure 1A; results for BUN were not recorded for 4 of the 10 control dogs). As most control dogs were not deemed to have kidney dysfunction by the veterinarian, several blood parameters were not collected for 4 of the 10 control dogs but they were collected for all 10 CKD dogs. The average creatinine level for control dogs was 0.7 ± 0.1 mg/dL (*n* = 6), whereas for CKD dogs it was 5.5 ± 0.8 (*p* < 0.05 vs. Con; and data presented as Mean ± SEM). The average value for albumin for controls was 3.0 ± 0.3 g/L (*n* = 6) and for CKD it was 2.5 ± 0.2 (ns vs. Con). The average value for calcium for controls was 9.3 ± 0.8 mg/dL (*n* =6) and for CKD it was 10 ± 0.5 (ns vs. Con). The average phosphorus value for controls was 5.5 ± 0.9 mg/dL (*n* = 6) and for CKD it was 15.5 ± 2.2 (*p* < 0.05 vs. Con).

Histopathology: To further characterize the dogs into control and CKD groups, we also examined the H&E-stained renal sections for morphological differences in the renal corpuscle in all dogs (Figure 1B) There was a significant decrease in the average number of healthy glomeruli in CKD dogs (36 ± 5.01 %; *n* = 10) compared with control dogs (66.9 ± 4.5 %; *n* = 10; *p* < 0.05; Figure 1C). Taken together with increased circulating levels of BUN, we grouped the dogs into control or CKD to assess differences in circulating miRNA gene expression.

**Figure 2:**
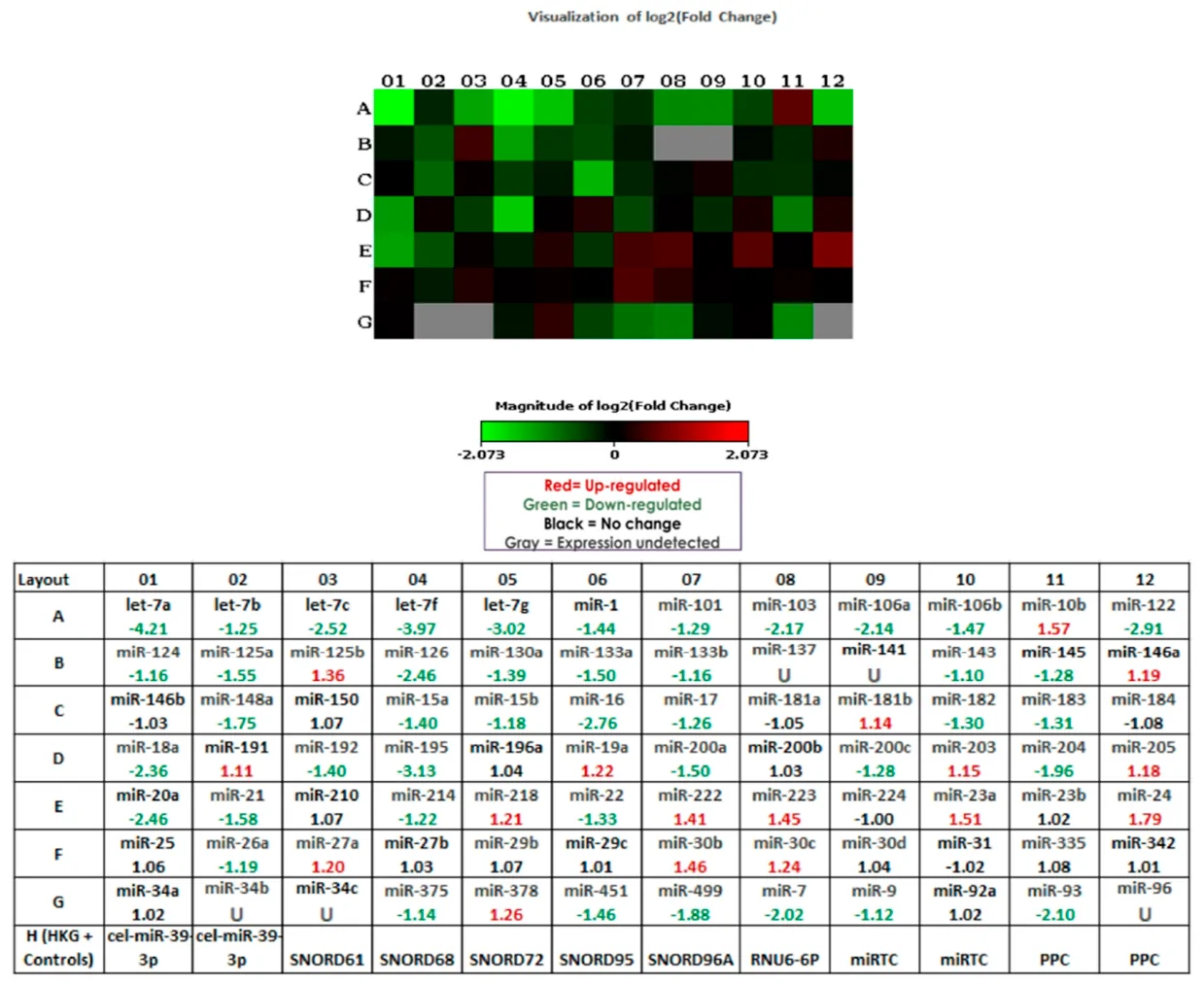
Heat map showing the fold change in gene expression in CKD dogs (*n* = 10) compared with controls (*n* = 10). The individual squares in a heat map are scaled with a range of colors proportional to gene expression values. The figure below (Rows A to G) corresponds to the squares above and each square indicates the average values for that gene, with numerically positive values indicating up-regulation and negative values indicating down-regulation. Row H includes the housekeeping genes SNORD61, SNORD68, SNORD72, SNORD95, and RNU-6P in the assay. Other controls in row H include a reverse transcription control and a positive PCR control. Data are presented as mean fold change (ΔΔCt) normalized to the housekeeping gene, SNORD68.

miRNA gene expression profile: The canine miRNA panel consisted of 84 genes and also several genes from the small nucleolar RNAs, C/D box (SNORD) family, including SNORD61, SNORD68, SNORD72, SNORD95, SNORD96A, and RNU6-6P (U6 small nuclear 6, pseudogene) that could be used as housekeeping genes. For this study, the data were normalized to SNORD68. This was chosen on the basis of the Ct values for both Control and CKD for each of the HKGs. Briefly, the Ct values ranged from 22.04 to 24.67 for SNORD68. For other HKGs, the Ct value ranges were: SNORD 61 (24–28), SNORD 72 (25.31–31.97), SNORD 95 (24.5–27.41), SNORD 96A (25.75–29.43), and RNU-6P (24.27–27.99). Of the 84 genes assessed, there was a decrease in 43 miRNAs, an increase in 17 miRNAs, and little to no change in 19 miRNAs and 5 were undetermined in the CKD group compared with controls (Figure 2).

**Figure 3:**
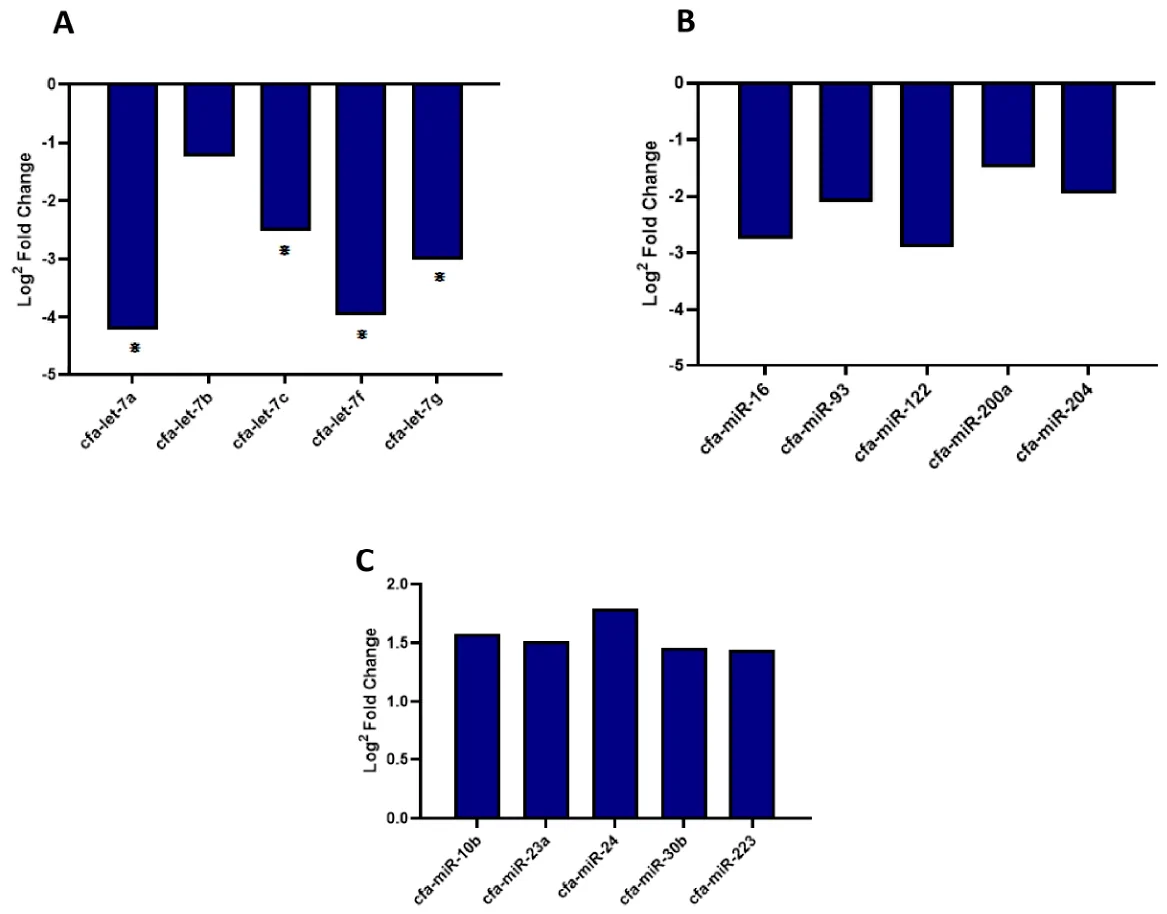
Graphical representation of selected miRNAs that were down-regulated or up-regulated in dogs with chronic kidney disease (CKD; *n* = 10) compared with controls (*n* = 10). (**A**) There was a significant down-regulation in *cfa*-let-7a, *cfa*-let-7c, *cfa*-let-7f, and *cfa*-let-7g in CKD dogs compared with controls (* *p* < 0.05). *cfa*-let-7b decreased but was not statistically significant. (**B**) Other miRNAs that were down-regulated (≥1.5-fold reduction) include *cfa*-miR-16, *cfa*-miR-93, *cfa*-miR-122, *cfa*-miR-200a, and *cfa*-miR-204. (**C**) miRNAs that were upregulated (>1.4 fold) in CKD dogs compared with controls (ns). Data are presented as mean fold change (ΔΔCt) normalized to SNORD68, the housekeeping gene. Fold-change values greater than one indicate an up-regulation. Fold-change values less than one indicate a negative or down-regulation, and the fold-regulation is the negative inverse of the fold-change.

There was a significant decline in the levels of *cfa*-let-7 miRNAs, including *cfa*-let-7a (−4.21 fold), *cfa*-let-7c (−2.52 fold), *cfa*-let-7f (−3.97 fold), and *cfa*-let-7g (−3.02 fold) in dogs with CKD compared with controls (all *p* < 0.05 vs. Con, Figure 3A). The decline in *cfa*-let-7b was modest but not statistically significant (−1.25 fold). There was also a ≥ 1.5-fold reduction in levels of *cfa*-miR-16 (−2.76 fold), *cfa*-miR-93 (−2.10 fold), *cfa*-miR-122 (−2.91 fold), *cfa*-miR-200a (−1.5 fold), and *cfa*-miR-204 (−1.96 fold), in CKD compared with controls (all ns vs. Con, Figure 3B). In contrast, there was an increase in several circulating miRNAs in CKD, but none were statistically significant compared with controls, including *cfa*-miR-10b (1.57 fold), *cfa*-miR-23a (1.51 fold), *cfa*-miR-24 (1.79 fold), *cfa*-miR-30b (1.46 fold), and *cfa*-miR-223 (1.45 fold; Figure 3C).

## 4. Discussion

Our results show a significant down-regulation of selected *cfa*-let-7 miRNAs (*cfa*-let-7a, *cfa*-let-7c, *cfa*-let-7f, *cfa*-let-7g) in dogs with CKD. This is consistent with human studies where a decrease has been reported in levels of let-7a-5p [26] and let-7c [27] in patients with advanced levels of CKD, as well as a decrease in let-7g-5p in CKD patients with hypertension [28]. In addition, inflammation is a key feature of CKD [29,30] and a reduction in selected let-7 miRNAs may also contribute to inflammation. Ye et al. [31] reported that in primary renal tubular epithelial cells, when let-7a was overexpressed or the PI3K/Akt signaling pathway was inhibited, the LPS-induced inflammatory response was attenuated. Upregulating let-7f in RAW264.7 macrophages through a traditional Chinese medicine ingredient has been shown to reduce the LPS-induced inflammatory response by inhibiting the TLR4 pathway [32]. In patients with lupus nephritis, Tangtanatakul et al. [33] reported a downregulation of -let-7a as well as miR-21 in urine exosomes during disease flare, but the levels of these miRNAs were elevated following treatment. These studies indicate that increasing levels of select let-7 miRNAs may play a beneficial role in attenuating impaired renal function. However, it also appears that the profile of miRNAs in impaired kidney function may be different when assessed in plasma, exosomes, or tissue. Lee et al. [34] reported a differential expression pattern of Let-7 miRNAs in a small set of patients who underwent kidney transplantation, where ischemia/reperfusion injury is generally observed. They found an upregulation of miR-let-7a-3p in postperfusion plasma along with an increase in miR-143-3p and miR-214-3p. However, let-7d-3p along with miR-1246, miR-1260b, miR-1290, and miR-130b-3p were downregulated. The profiling of miRNAs, including let-7 miRNAs, appears to be complex, as they may depend on the type of kidney dysfunction and/or injury, as well as the matrix where it is measured. Further studies are warranted to validate the role of circulating miRNAs in naturally occurring impaired kidney function. More importantly, the identification of let-7 might serve as a therapeutic target. While cell-based assays are currently under investigation to augment levels of functional mature let-7 miRNAs [35], the role of nutrition to target these miRNAs in CKD might serve to complement important therapeutic strategies. Table 1 highlights some of the target genes for selected let-7 miRNAs in renal dysfunction or pathology. While the subsequent functional effects may depend on whether let-7 levels are upregulated or downregulated in the studies cited, it is imperative to further investigate the specific targets of let-7 miRNAs as related to naturally occurring impaired kidney function in canines. Furthermore, since a single miRNA can have multiple mRNA targets or any mRNA can be a target of multiple miRNAs [36], further studies will assess the mRNA targets of these miRNAs associated with kidney dysfunction.

Other miRNAs that showed a reduction, although not statistically significant, in the present study include *cfa*-miR-16, *cfa*-miR-93, *cfa*-miR-122, *cfa*-miR-200a, and *cfa*-miR-204, in CKD compared with controls. Several of these miRNAs have been reported to have an anti-fibrogenesis effect and are also down-regulated in rodent models and in vitro mechanistic models of renal fibrosis, leading to increased fibrosis. Renal fibrosis is a key feature of kidney injury and impaired kidney function in CKD [48,49] and is characterized by normal tissue being replaced over time by scar tissue, leading to eventual kidney dysfunction. While the role of miR-16 in renal fibrosis is not clear, Yao et al. [50] reported that miR-16 was downregulated in patients with systemic sclerosis, a condition characterized by fibrosis. Using an in vitro model, they showed that miR-16 could bind directly to NOTCH-2, an activator of extracellular matrix accumulation, to inhibit myofibroblast activation and subsequent collagen expression. Whether increasing levels of miR-16 are beneficial in renal dysfunction needs to be investigated. It is conceivable that a decrease in miR-16 is one factor that could lead to increased fibrosis. If true, then EGCG, a polyphenol from green tea, has been shown to upregulate miR-16 in human cancer cells [51], indicating that dietary nutritional components have the potential to increase miR-16. In contrast, Zhu et al. [52] reported upregulation of miR-16 that was associated with increased fibrosis, likely due to its actions on HGF and Smad7. Similarly, Pan et al. [53] reported a role for miR-16 in resolving liver fibrosis using miR-16 indicating that attenuating miR-16 is beneficial for liver fibrosis. It should be noted that, in our study, the reduction in *cfa*-miR-16 is not statistically significant and further studies may be warranted to establish its role in fibrosis. 

In addition, there was also a decrease in selected miRNAs in CKD dogs that are important in the development and/or progression of renal fibrosis, including miR-93 and miR-200a. Ma et al. [54] showed that miR-93 was reduced in renal tissue of patients with diabetic nephropathy. They further showed that overexpression of miR-93 in TGF-b stimulated HK-2 cells suppressed TGF-β1-mediated EMT and fibrogenesis. Wang et al. [55], using cell culture as well as mouse models of diabetic nephropathy, reported that miR-200a was involved in the regulation of TGF-β-dependent fibrosis, likely through downregulation of TGF-β2. A decrease in both miR-93 and miR-200a in the present study could then contribute to increased fibrosis, although both these miRNAs did not reach statistical significance. However, it is also possible that the decrease seen in miR-200a was a response to tubular stress or injury. Limited evidence in human studies appears to indicate that attenuating miR-200a might even have a beneficial effect on kidney function. Trabulus et al. [56] reported an increase in miR-200a in urine from patients with diabetic kidney disease and also those with focal segmental glomerulosclerosis. Martinez-Arroyo et al. [57] also reported an increase in miR-200a in hypertensive patients associated with diabetes. They then used in vitro models using renal tubular cells and showed that inhibiting miR-200a reduced tubular damage induced by high glucose and angiotensin II, indicating that inhibition of miR-200a was beneficial. Whether there would be differences in the expression of these miRNAs between species even when assessing renal dysfunction due to stress or injury is not clear but is a possibility. miRNAs can also have a dual role where the miRNA may be involved in protective or deleterious effects. For instance, administration of miR-23a in diabetic mice attenuated renal fibrosis through its action on Akt and FoxO1 signaling [58], whereas Li et al. [59] reported that increased miR-23a activated macrophages and subsequent pro-inflammatory cytokine levels. In the present study, we observed an increase in miR-23a in CKD dogs compared with control dogs. There was also an increase in levels of miR-10b, miR-23a, miR-24, miR-30b, and miR-223 in CKD dogs. While the increases were not statistically significant, the combined biological effect of the increase in these miRNAs on a biological function is a possibility. Wang et al. [60] reported that miR-10a and miR-10b were overexpressed in a mouse model of unilateral ureteral obstruction (UUO) and knockout of these miRNAs attenuated renal fibrosis in UUO-treated mouse kidneys. Similarly, Shuai et al. [61], using a mouse model of UUO, reported increased expression of miR-10a and miR-10b in the kidney of UUO mice. Further, using a cell culture model of TGF-β1 stimulation in HK-2 cells, they reported that inhibiting these miRNAs resulted in a decrease in markers of fibrosis. These studies provide a strong rationale to investigate further the role of miRNAs in canine CKD and to investigate whether profiling circulating miRNAs would help in the assessment of impaired kidney function.

Taken together with the literature, down-regulation of selected miRNAs may be indicative of a reduction in their role in attenuating renal fibrogenesis and injury. Nevertheless, it is important to consider that several of these miRNAs can also have dual effects, including beneficial and deleterious effects.

One limitation of our study is the sample size of 10 dogs in each group, which could be increased in future studies with a more controlled range of age. While in naturally occurring CKD in dogs, like in our study, it may pose a challenge to selectively choose the age range, it still needs to be included as an important component in the selection of CKD dogs in the future. Further, our data should be interpreted with caution as more targeted qPCR for further validation needs to be conducted in the future. 

## 5. Conclusions

Identifying circulating miRNAs associated with impaired kidney function may provide useful information to detect initiation and/or progression of renal dysfunction. While our study provides a rationale for assessing the role of circulating miRNAs as non-invasive biomarkers for impaired renal function, their role in renal fibrosis in canine CKD needs to be assessed in future studies. Whether these circulating miRNAs may serve as attractive nutritional targets for slowing the progression of kidney disease is not clear but it is a possibility that needs to be investigated.

## Figures and Tables

**Table 1:** Some mRNA targets of selected let-7 miRNAs in renal dysfunction or pathology.

miRNA	Selected Targets (mRNA)	Condition/Disease	Reference/Pathway
let-7a	RRM2	diabetic kidney disease (DKD)	[37]
	CDKN1A, IL-6, MYC	membranous glomerulonephritis	[38]
	TNF-α, IL6, IL-1β	Acute kidney injury	[31]
let-7b	TGF-βR1	renal fibrosis	[39]
	GALNT2	IgA nephropathy	[40]
	TGF-βR1, ARID3A	renal fibrosis	[41]
	TGF-βR1, COL1A1, H19	tubular epithelial fibrosis	[42]
let-7c	TGF-β1	chronic kidney disease (CKD)	[27]
	TGF-β1, TGF-βR1	renal Fibrosis	[43]
	IL-6, MYC	membranous glomerulonephritis	[38]
	AKT2, renin, CD36	Renal cell carcinoma; hypertensive kidney	[44,45]
let-7f	THBS1	renal fibrosis	[46]
	IL-10, TGF-β1, CST3	lupus nephritis, systemic lupus erythematosus	[47]
let-7g	COL1A2, FN1, SMAD2, THBS1	renal fibrosis	[46]

Abbreviations: AKT Serine/Threonine Kinase 2 (AKT2); AT-Rich Interaction Domain 3A (ARID3A); Cluster of Differentiation 36 (CD36); Collagen Type I Alpha 1 Chain (COL1A1); Collagen Type I Alpha 2 Chain (COL1A2); Cystatin C (CST3); Fibronectin 1 (FN1); GRB2 Associated Binding Protein 1 (GAB1); Polypeptide N-Acetylgalactosaminyltransferase 2 (GALNT2); H19 Imprinted Maternally Expressed Transcript (H19); Hepatocyte Growth Factor (HGF); HRas Proto-Oncogene, GTPase (HRAS); Interleukin 1 Beta (IL-1β); Interleukin 6 (IL-6); Interleukin 6 Receptor (IL-6R); Interleukin 10 (IL-10); Insulin Like 3 (INSL3); Janus Kinase 3 (JAK3); KRAS Proto-Oncogene, GTPase (KRAS); Lin-28 Homolog A (LIN28A); Mitogen-Activated Protein Kinase 1 (MAP2K1); Mitogen-Activated Protein Kinase 2 (MAP2K2); MYC Proto-Oncogene, BHLH Transcription Factor (MYC); NRAS Proto-Oncogene, GTPase (NRAS); P21 (RAC1) Activated Kinase 1 (PAK1); Platelet Derived Growth Factor Subunit B (PDGFB); Ribonucleotide Reductase Regulatory Subunit M2 (RRM2); SMAD Family Member 2 (SMAD2); Transforming Growth Factor Beta 1 (TGF- β1); Transforming Growth Factor Beta Receptor 1 (TGF-βR1); Thrombospondin 1 (THBS1); Tumor Necrosis Factor (TNF-α).

## Data Availability

Currently the datasets presented in this article are not readily available because the data are part of an ongoing study. Moreover, data may be made available, if approved internally by, Hill’s PNC (A Colgate-Palmolive company). Requests to access the datasets should be directed to the corresponding author.
